# Supplementary material for: Relationship between the skeletal muscle mass index and physical activity of Japanese children: A cross-sectional, observational study
Source: PLoS One. 2021 May 26;16(5):e0251025. doi: 10.1371/journal.pone.0251025 (PMC8153420; doi:10.1371/journal.pone.0251025)
Supplement: S1 Table — (DOCX) [file pone.0251025.s001.docx]

**S1 Table. Demographic Characteristics and Physical Function Outcomes of Study Participants.**

| **Variable** | **Boys (*n*=165)** | **Girls (*n*=175)** | ***P* value^*^** | **Effect size (*r*)** |
| --- | --- | --- | --- | --- |
| **Age (years)^a^** | 10.0 (6.0–12.0) | 9.0 (6.0–12.0) | 0.803 | -0.01 |
| **Height (cm)^a^** | 136.0 (106.5–164.2) | 135.0 (108.8–163.6) | 0.328 | -0.1 |
| **Weight (kg)^a^** | 30.7 (16.1–74.4) | 29.0 (16.6–51.9) | 0.231 | -0.1 |
| **Body mass index (kg/m^2^)^a^** | 15.7 (13.13–29.6) | 15.87 (12.32–23.57) | 0.481 | -0.04 |
| **Skeletal muscle mass index (kg/m^2^)^a^** | 6.01 (4.64−8.48) | 5.63 (4.55−6.93) | 0.0001 | -0.3 |
| **Percent body fat (%)^a^** | 10.5 (5.0–46.4) | 14.2 (5.8–34.8) | 0.0001 | -0.3 |
| **Gait Deviation Index (points)^a^** | 94.3±7.69 | 94.6±6.98 | 0.685 | 0.02 |
| **Gait speed (m/s)^a^** | 1.19±0.17 | 1.17±0.16 | 0.282 | 0.1 |
| **Grip strength (kg)^a^** | 13.55 (5.9−33.0) | 12.75 (5.75−26.8) | 0.044 | -0.1 |
| **Five Times Sit-to-Stand Test (s)^a^** | 6.03 (3.28−11.35 | 6.06 (4.74−10.6) | 0.779 | -0.02 |
| **Timed Up-and-Go test (s)^a^** | 7.47 (4.57−11.57) | 7.47 (4.74−10.98) | 0.685 | -0.02 |
| **One-leg standing time (s)^a^** | 110.0 (2.64−120.0) | 120 (6.9−120.0) | 0.002 | -0.2 |
| **Physiological cost index (beat/m/s)^a^** | 21.8 (0−76.29) | 23.46 (0−57.61) | 0.231 | -0.1 |
| **MVPA times per week (h)^a^** | 7.0 (0–25.5) | 4.0 (0–28) | 0.109 | -0.1 |

^a^Data are presented as means±standard deviations or medians (ranges).

^*^*P* values for the Gait Deviation Index and gait speed were derived using an independent t-test. For all other variables, *P* values were derived using the Mann–Whitney U test.

MVPA, moderate-to-vigorous physical activity
