# Supplementary material for: Relationship between the skeletal muscle mass index and physical activity of Japanese children: A cross-sectional, observational study
Source: PLoS One. 2021 May 26;16(5):e0251025. doi: 10.1371/journal.pone.0251025 (PMC8153420; doi:10.1371/journal.pone.0251025)
Supplement: S2 Table — (DOCX) [file pone.0251025.s002.docx]

**S2 Table**. **Demographic Seasonal Variations in Physical Activity of the Participants** **(*n*=340).**

| **Variables** | **Spring**  **(*n=*93)** | **Summer**  **(*n*=70)** | **Autumn**  **(n=78)** | **Winter**  **(*n*=99)** | ***P*-value** | **Effect size (*η^2^*)** |
| --- | --- | --- | --- | --- | --- | --- |
| **MVPA times per week (h)** | 7.0 (0–21.0) | 6.0 (0–20.0) | 4.0 (0–20) | 3.8 (0–28) | 0.061 | 0.02 |

Data are presented as medians (ranges).

Differences between the four seasons and the physical activity time were analyzed using one-way analysis of the Kruskal-Wallis test. However, the physical activity levels were not significantly different because of the seasonal variations in this study (*P*=0.061; η^2^=0.02), indicating that these seasonal variations observed in the winter vs. summer/spring months did not necessarily exhibit significant changes in time dedicated to physical activity.

MVPA, moderate-to-vigorous physical activity
