# Supplementary material for: Relationship between the skeletal muscle mass index and physical activity of Japanese children: A cross-sectional, observational study
Source: PLoS One. 2021 May 26;16(5):e0251025. doi: 10.1371/journal.pone.0251025 (PMC8153420; doi:10.1371/journal.pone.0251025)
Supplement: Attachment — Submitted filename: Certificate_of_editing.pdf [file pone.0251025.s005.pdf]

Editing Certificate

This document certifies that the manuscript listed below has been edited to ensure language and grammar accuracy and is error free in these aspects. The logical presentation of ideas and the structure of the paper were also checked during the editing process. The edit was performed by professional editors at Editage, a division of Cactus Communications. The author's core research ideas were not altered in any way during the editing process. The quality of the edit has been guaranteed, with the assumption that our suggested changes have been accepted and the text has not been further altered without the knowledge of our editors.

MANUSCRIPT TITLE

Relationship between the skeletal muscle mass index and physical activity of Japanese children: A cross-sectional, observational study

AUTHORS

Tadashi Ito

ISSUED ON

March 30, 2021

JOB CODE

ADTOZ_11_18

THE EDITAGE Promise QUALITY GUARANTEE

Vikas Narang

Vikas Narang
Chief Operating Officer - Editage

editage

Editage, a brand of Cactus Communications, offers professional English language editing and publication support services to authors engaged in over 1300 areas of research. Through its community of experienced editors, which includes doctors, engineers, published scientists, and researchers with peer review experience, Editage has successfully helped authors get published in internationally reputed journals. Authors who work with Editage are guaranteed excellent language quality and timely delivery.

GLOBAL : (833) 979-0061 | request@editage.com

JAPAN : 0120-50-2987 | submissions@editage.com

CACTUS

IMPACT SCIENCE impact.science

researcher.life

CACTUS LIFE SCIENCES lifesciences.cactusglobal.com
