# Supplementary material for: Relationship between the skeletal muscle mass index and physical activity of Japanese children: A cross-sectional, observational study
Source: PLoS One. 2021 May 26;16(5):e0251025. doi: 10.1371/journal.pone.0251025 (PMC8153420; doi:10.1371/journal.pone.0251025)
Supplement: Attachment — Submitted filename: Reveiewers_Response_PLOSONE_20210212.docx [file pone.0251025.s004.docx]

February 12, 2021

Joerg Heber

Editor-in-Chief

*PLOS ONE*

Dear Editor:

We would like to thank you for your response and for giving us the opportunity to improve and resubmit our manuscript (ID: PONE-D-20-38059) entitled "**Relationship between skeletal muscle mass index and physical activity of Japanese children: A cross-sectional, observational study**." We are hereby resubmitting a revised manuscript conforming to all of the reviewers’ comments. In particular, we have addressed all the reviewers’ comments in a point-by-point manner and revisions are indicated in red font in the revised paper We hope that the revised manuscript is now suitable for publication in your journal.

Thank you for your consideration. I look forward to hearing from you.

Sincerely,

Tadashi Ito

Three-dimensional Motion Analysis Room

Aichi Prefectural Mikawa Aoitori Medical and Rehabilitation Center for Developmental Disabilities

9-3 Koyaba Kouryuji cho, 444-0002 Okazaki, Japan

Tel: +81-564-64-7980

Fax: +81-564-64-7981

Email: [sanjigen@mikawa-aoitori.jp](mailto:sanjigen@mikawa-aoitori.jp)

**Editor #1**

**Comments**

Journal Requirements:

When submitting your revision, we need you to address these additional requirements.

1. Please ensure that your manuscript meets PLOS ONE's style requirements, including those for file naming. The PLOS ONE style templates can be found at

https://journals.plos.org/plosone/s/file?id=wjVg/PLOSOne_formatting_sample_main_body.pdf and

https://journals.plos.org/plosone/s/file?id=ba62/PLOSOne_formatting_sample_title_authors_affiliations.pdf

**Response:** The authors would like to thank the Editor for the constructive critique to improve the manuscript. We have made every effort to address the issues raised and to respond to all comments. The revisions are indicated in red font in the revised manuscript.

**Comments**

2. Thank you for stating the following financial disclosure:

'The funders had no role in study design, data collection and analysis, decision to publish, or preparation of the manuscript.'

At this time, please address the following queries:

Please clarify the sources of funding (financial or material support) for your study. List the grants or organizations that supported your study, including funding received from your institution.

State what role the funders took in the study. If the funders had no role in your study, please state: “The funders had no role in study design, data collection and analysis, decision to publish, or preparation of the manuscript.”

If any authors received a salary from any of your funders, please state which authors and which funders.

If you did not receive any funding for this study, please state: “The authors received no specific funding for this work.”

Please include your amended statements within your cover letter; we will change the online submission form on your behalf.

**Response:** We would like to thank the Editor for the comment. Please note that we have not received any specific funding for this work. This information has been included and clarified in the cover letter and manuscript files in accordance with the Editor’s comment.

**Reviewer #1**

The authors would like to thank the reviewer for his/her constructive critique to improve the manuscript. We have made every effort to address the issues raised and to respond to all comments. The revisions are indicated in red font in the revised manuscript. Please, find next a detailed, point-by-point response to the reviewer's comments.

**Comment**

Thank you for the hard-effort observational study. This study has tried to reveal the relationship between sufficient PA and skeletal muscle mass in growing children. And even more, authors tried to figure out the real physical performance by examining several time-consuming studies such as TUGT, one-leg standing time, sit to stand test results, grip strength, gait, etc.

**Response:** We would like to thank the reviewer for the positive evaluation of our work.

**Comment**

Method

Please define when skeletal muscle mass assessment with BIA was done as the previous food, or liquid consumption could influence the results.

**Response:** We would like to thank the reviewer for the constructive comment. Please note that we have added this information to the sentence describing the skeletal muscle mass assessment with BIA, as per the reviewer’s suggestion.

“Bioelectrical impedance analysis (BIA) was only performed at 2 h after the meals, as described in the instruction manual.” (Lines 145–147)

**Comment**

Would you please describe details about the grip strength? Did you have a practice trial or not? How many times did the participants evaluate?

**Response:** We would like to thank the reviewer for pointing this out. Grip strength was measured once each to the right and left limbs. Moreover, the participants were allowed to perform one trial, as a learning attempt. We have amended this information in the Methods section, as follows: “The dominant hand was measured first, followed by the non-dominant hand. The grip strength of both hands was measured once each with the participants in the sitting position with their shoulder abducted and neutrally rotated, the elbow at extension, and the forearm and wrist in the neutral position [28]. The participants were instructed to squeeze the handle of the dynamometer as hard as they could and sustain for 5 s [29]. Verbal encouragement (i.e., “Keep it up, keep it up”) was provided during the test [29]. Prior to the grip strength assessment by an experienced physical therapist, the participants were allowed to perform one practice trial as a learning attempt. The grip strength measurement reliability was reported to be excellent in a full elbow-extension position [30].” (Lines 173–181)

**Comment**

What do you mean “The funder played no role in the design, conduct, or reporting of this study.” It is written that the manuscript has no funding resources.

**Response:** We would like to thank the reviewer for the comment. Please note that we have received any specific funding for this work. This error has been corrected in accordance with the reviewer’s comment.

**Comment**

Statistical analysis and results

Do you have any special reason for comparing median age, height, and weight? It seems a sufficient number to compare with t-test.

**Response:** We would like to thank the reviewer for providing these insightful comments. The t-test cannot be performed because the age, height, and weight data were not normally distributed.

**Comment**

Are there any age-specific characteristics in the pattern of MVPA levels? I wonder if they had a different percentage of sufficient MVPA level according to the age group. With the supplementary data, I realized that a relatively small number of 6-aged students and they all are almost in suboptimal PA group, for example.

**Response:** We would like to thank the reviewer for pointing this out. The percentages of children that performed the recommended MVPA level according to their age groups were as follows: 6-year-old, 11.8%; 7-year-old, 25.0%; 8-year-old, 31.6%; 9-year-old, 37.7%; 10-year-old, 57.6%; 11-year-old, 72.3%; and 12-year-old, 53.0%. With respect to this study characteristic, we presumed that children aged 10–12 actively participated in school clubs, and the opportunities for participation in sports activities are numerous. We have provided this information in the Results section.

**Comments**

It would be better to demonstrate a general logistic regression table instead of a simple P-value and 95% CI.

**Response:** We would like to thank the reviewer for the comment. We agree with the reviewer’s suggestion. Therefore, we have included this information in Table 3.

**Comment**

Discussion

Recommend some of the points to discuss.

One study revealed that disability significantly affects muscle mass. (Pediatr Int. 2020 Aug;62(8):962-969. doi: 10.1111/ped.14248.)

Another study figured out that PA is also associated with positive mental health in a multi-ethnic Asian population. (Int J Environ Res Public Health. 2020 Nov 16;17(22):8489. doi: 10.3390/ijerph17228489.)

**Response:** We would like to thank the reviewer for the suggestion. We agree that the points mentioned need to be added to the Discussion section. Following the reviewer's insightful suggestion, we have revised these sentences in the Discussion section and cited the relevant literature, as follows:

“Moreover, one study revealed that disability significantly affected the muscle mass [47].” (Lines 313–314)

“Another study reported that PA was also associated with positive mental health in a multi-ethnic Asian population [65]. We presumed that children in our study actively participated in school clubs, and the opportunities for participation in sports activities are numerous.” (Lines 358–361)

**Reviewer #2**

We would like to thank the reviewer for providing insightful comments regarding our manuscript. We express our sincere gratitude for the time and effort the reviewer has spent to review our manuscript. The comments and suggestions have helped us immensely improve our work.

**Comments**

Overall Impressions: Well done study. Statistical analysis appears appropriate. Sample size appears appropriate. Some limitations in methodology need to be addressed.

**Response:** We would like to thank the reviewer for the positive comments regarding our study. The manuscript has been rechecked, and the necessary changes have been made in accordance with the reviewers’ suggestions. The responses to all comments have been prepared and given below.

**Comments**

a) Please report on the reliability and precision of muscle function and physical performance tests. Should provide some evidence of this to ensure tests were reliable.

**Response:** We agree with the reviewer’s comment. Therefore, we have provided this information in the Materials and Methods section (Assessment of appendicular skeletal muscle mass, Grip strength, Five times sit-to-stand test, Timed Up-and-Go test, One-leg standing time, and Gait efficiency).

**Comments**

b) Use of BIA to assess appendicular skeletal muscle mass is impacted by normative equations used by equipment. Authors need to address the limitations of BIA in the discussion and to report on precision and reliability data.

**Response:** Following the reviewer’s suggestion, we have added more information to the Discussion section as follows: “Fourth, the validity of the BIA method depends on the participant’s hydration status. Limitations of BIA include assumptions involving a fixed hydration status [67]. Thus, BIA may not always be the best method to assess body composition.” (Lines 375–377)

**Comments**

c) Use of subjective measures of PA (questionnaire) is limited and may have impacted overall study findings. It would be best to explore the extent to which bias could have impacted overall study findings in discussion. This should be expanded in more detail in the discussion as this would have limited overall study findings.

**Response:** We would like to thank the reviewer for pointing this out. We agree with the reviewer’s comment. Therefore, we have added the following part to the Discussion section:

" However, the categorization of MVPA relied on self-reported questionnaire data that could be affected by recall bias. Subjective assessment was reported to overestimate MVPA compared with objective assessment [66]. Moreover, as the items for measuring preference for MVPA were not investigated for reliability and validity, a future longitudinal study is needed to confirm this hypothesis.” (Lines 364–368)

**Comments**

d) Making the assumption of physical activity outside of ‘school hours’ may not be appropriate and hence the authors should temper their overall conclusions on this point.

**Response:** We agree that the assumption of physical activity outside of “school hours” needs improvement. Therefore, we have revised the corresponding part in the manuscript to avoid its misinterpretation as follows: “We presumed that children in our study actively participated in school clubs, and the opportunities for participation in sports activities are numerous.” (Lines 360–361)

**Comments**

e) Any seasonal variations in physical activity noted? This would be important to include as school aged children PA patterns may vary substantively over the winter vs summer/spring months.

**Response:** We would like to thank the reviewer for the suggestion. Differences between the four seasons and the physical activity time were examined using one-way analysis of the Kruskal–Wallis test. However, the physical activity levels were not significantly different because of the seasonal variations in this study (*P*=0.061; η^2^=0.02), indicating that these seasonal variations observed in the winter vs. summer/spring months did not necessarily exhibit significant changes in physical activity time. We agree that we should focus on the seasonal variations and, therefore, we have provided more information in S2 Table.
